# Fibromuscular dysplasia with recurrence after “long-term” following percutaneous transcatheter renal angioplasty: two case reports with a review of 26 patients

**DOI:** 10.1186/s12882-021-02342-w

**Published:** 2021-05-20

**Authors:** Shuntaro Oribe, Takafumi Toyohara, Eikan Mishima, Takehiro Suzuki, Koichi Kikuchi, Shun Watanabe, Yoshiaki Morita, Hideki Ota, Kazumasa Seiji, Mariko Miyazaki, Kei Takase, Takaaki Abe

**Affiliations:** 1 Division of Nephrology, Endocrinology, and Vascular Medicine, Tohoku University Graduate School of Medicine, Sendai, Japan; 2 Division of Medical Science, Tohoku University Graduate School of Biomedical Engineering, Sendai, Japan; 3 Department of Diagnostic Radiology, Tohoku University Graduate School of Medicine, Sendai, Japan; 4 Department of Clinical Biology and Hormonal Regulation, Tohoku University Graduate School of Medicine, 980-8574 Sendai, Japan

**Keywords:** Fibromuscular dysplasia, Percutaneous transcatheter renal angioplasty, Recurrence, Renovascular hypertension, Renal artery stenosis, Multifocal FMD, Focal FMD

## Abstract

**Background:**

Fibromuscular dysplasia (FMD) often causes renal artery stenosis with renovascular hypertension. Recent clinical outcomes encourage percutaneous transluminal renal angioplasty (PTRA) to treat FMD; however, the necessary follow-up period remains unclear. Moreover, previous studies have not revealed the difference in the period until recurrence between two major types of FMD—multifocal and focal.

**Case presentation:**

We describe two patients with multifocal FMD who developed hypertension during their teenage years and had recurrence of FMD > 10 years after PTRA. We further examined the types of FMD and age of onset in 26 patients who underwent PTRA. The period until recurrence of multifocal FMD was longer than that of focal FMD. Moreover, patients with early-onset multifocal FMD are likely to have a delayed recurrence after PTRA compared to other types.

**Conclusions:**

Our report suggests that patients with multifocal FMD, especially those with onset at an early age, may need long-term follow-up for at least ≥ 10 years.

## Background

Fibromuscular dysplasia (FMD) is an idiopathic, noninflammatory, and non-atherosclerotic vascular disease that often causes renovascular hypertension (RVH) due to renal artery stenosis (RAS) [1]. Percutaneous transluminal renal angioplasty (PTRA) is known to be effective and has been widely performed to treat RVH caused by FMD [2–4]. Although the recent international consensus on the diagnosis and management of FMD advocated studies on clinical outcomes of PTRA as one of the research priorities,[5] the necessary follow-up period after PTRA remains unclear. Only some studies have shown that RVH recurred usually within 5 years after PTRA [2, 6, 7]. FMD is categorized into two types, based on the angiographic appearances, as multifocal FMD and focal FMD [5, 8, 9]. These types might have different clinical courses after PTRA; however, the difference in recurrence period between the two types has not been investigated.

In this report, we present two patients who were followed up for a long period and showed recurrence more than 10 years after PTRA. Patient 1 was followed up for more than 30 years, during which she had two recurrences. We also examined the outcomes of 26 FMD patients treated with PTRA in our institute between 2008 and 2019 to determine which patients require a longer follow-up period and the difference in prognosis between focal FMD and multifocal FMD after PTRA.

## Case presentation

### Patient 1

**Fig. 1:**
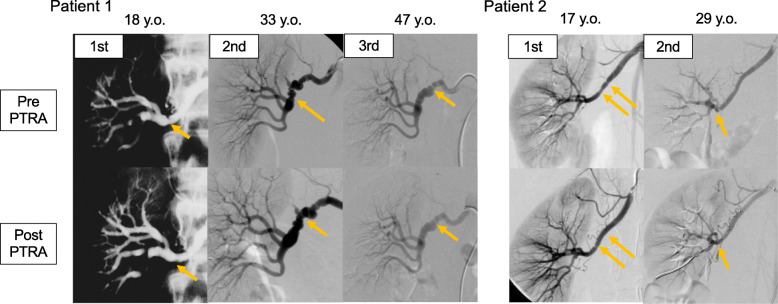
Angiographic images of patients 1 and 2. Angiographic images before and after PTRA (top: pre-PTRA, bottom: post-PTRA). Yellow arrows show stenotic lesions dilated by PTRA. PTRA: percutaneous transluminal renal angioplasty; y.o.: years old

A 47-year-old woman was admitted to our hospital for uncontrolled high blood pressure (BP). Her BP was 223/119 mmHg despite medication with nifedipine 40 mg. She had been diagnosed with RVH due to right-sided multifocal FMD and had undergone PTRA at the age of 18 years (Fig. 1). At the age of 34 years (16 years after the first PTRA), her BP increased again due to a recurrence of RAS in a different segment of the right renal artery, and she underwent a second PTRA (Fig. 1). After the second PTRA, follow-up was discontinued for several years as her BP remained normal. However, 13 years after the second PTRA, her BP increased again and she was referred to our hospital. Computed tomography (CT) revealed right RAS, and Doppler ultrasound showed a peak systolic velocity (PSV) of 238 cm/s and renal/aorta ratio (RAR) of 3.3, suggesting a second recurrence of RVH due to FMD. An angiography showed RAS at a different segment of the right renal artery, with a mean pressure gradient of 20 mmHg (Fig. 1). After the third PTRA, her BP returned to normal. Thus, the patient was followed up for a total of more than 30 years since initial onset.

### Patient 2

A 29-year-old woman was admitted to our hospital for hypertension. Her BP was 158/113 mmHg after treatment with amlodipine 2.5 mg. She had been diagnosed with RVH due to right-sided multifocal FMD and had undergone PTRA at the age of 17 years (Fig. 1). After PTRA, her BP remained normal without any antihypertensive medication. However, 12 years after PTRA, her BP increased to 130–140/90–100 mmHg. CT imaging suggested the recurrence of the right RAS due to multifocal FMD. Doppler ultrasound showed that the PSV was 402 cm/s and RAR was 4.8. By angiography, the diagnosis was recurrence of RVH, with a systolic pressure gradient of 86 mmHg (Fig. 1). After the second PTRA, she has been followed up for 5 years until now, without any antihypertensive medication. The patient has thus far been followed up for a total of 17 years since initial onset.

### Additional investigations

Both cases developed multifocal FMD at a young age, suggesting that the recurrence period after PTRA might be delayed depending on the age of initial onset and the type of FMD. We cross-sectionally reviewed the clinical characteristics, especially the age at diagnosis and the type of FMD, of 26 FMD patients who underwent PTRA in our hospital from 2008 to 2019.

**Fig. 2:**
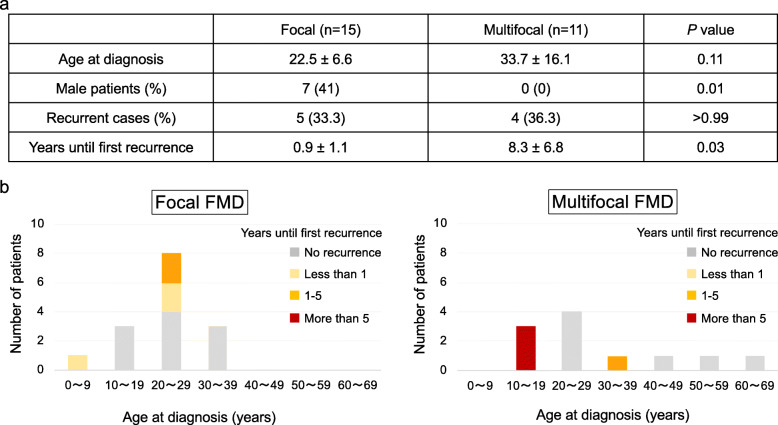
Comparison of the clinical characteristics between patients with focal FMD and multifocal FMD. **a** Clinical data of patients with FMD who underwent PTRA in our institute between 2008 and 2019. **b** Distribution of patients depending on the age at diagnosis and type of FMD. The chart is color-coded by the years until the first recurrence after PTRA. No recurrence (gray), less than 1 year (yellow), 1 year or more and less than 5 years (orange), and more than 5 years (red). All three patients who had a recurrence after more than 5 years had multifocal FMD, and the age at onset age was within the teenage years. Data were compared using a Mann-Whitney test. Nominal variables were compared using a Fisher’s exact test. Data are represented as mean ± SD. *P* values < 0.05 were considered significant. FMD: fibromuscular dysplasia; PTRA: percutaneous transluminal renal angioplasty

As shown in Fig. 2 A, the age at diagnosis of multifocal FMD was slightly higher than that of focal FMD (multifocal FMD: 33.7 ± 16.1 years old vs. focal FMD: 22.5 ± 6.6 years old, respectively, *p* = 0.11; Fig. 2 a), and the proportion of male patients with focal FMD was higher (41 % vs. 0 %, *p* = 0.01; Fig. 2 a), which is similar to a previous report [11]. However, although the rate of recurrence was not different between the two types (multifocal FMD: 36.3 % vs. focal FMD: 33.3 %, *p* = > 0.99), the period until the recurrence of multifocal FMD was longer than that of focal FMD (multifocal FMD: 8.3 ± 6.8 years vs. focal FMD: 0.9 ± 1.1 years, *p* = 0.03). Figure 2b shows the detailed distribution of patients based on the age at diagnosis and type of FMD. All three patients who had recurrence more than 5 years after PTRA had multifocal FMD, including the two cases described above (red bar in Fig. 2b on the right). Three of five focal FMD patients (yellow bars in Fig. 2b on the left) had recurrence within 1 year; however, no recurrence within 1 year was seen in patients with multifocal FMD. These results suggest that the period until recurrence is likely longer in multifocal FMD than in focal FMD. In addition, multifocal FMD patients with a younger age of onset may especially have a longer period until recurrence, indicating that they need to be carefully followed up after PTRA, for at least more than 10 years.

Previous reports showed that the risk factors of recurrence are aging, past or current smoking status, low estimated glomerular filtration rate (eGFR), and high triglyceride (TG) level, and high-density lipoprotein cholesterol (HDL) level 6,10. We investigated whether these risk factors affected the recurrences in our cases. We noted no significant differences in the risk factors between patients with and without recurrences (smoking rate: 22.2 % vs. 24.0 %, *p* = > 0.99; eGFR: 97.2 ± 13.1 mL/min/1.73m2 vs. 93.1 ± 12.6 mL/min/1.73m2, *p* = 0.48; TG: 88.8 ± 40.0 mg/dL vs. 89.4 ± 25.0 mg/dL, *p* = 0.97; HDL: 56.4 ± 14.5 mg/dL vs. 62.5 ± 15.7 mg/dL, *p* = 0.36).

## Discussion and conclusions

To our knowledge, this is the first report describing patients with FMD who developed recurrence more than 10 years after PTRA. Especially, patient 1 was observed for 30 years since the first PTRA. This report suggests that the period until recurrence in multifocal FMD was likely to be longer than that in focal FMD; this has not been discussed in previous studies.

Our report indicates that the follow-up strategy for focal FMD and multifocal FMD should be different. Previous research showed that most recurrences of RVH occur within 5 years after PTRA [6, 7]. Only one study in which FMD patients were observed for 10 years after PTRA reported patients with BP elevation after more than 5 years [2]. These results are consistent with the recent international consensus on diagnosis and management of FMD that recommended surveillance with renal artery duplex ultrasound every 6 months for 2 years and then yearly to detect restenosis [5]. However, while patients with focal FMD should be observed as recommended in the consensus as most recurrences occur within the short term, a different approach might be necessary for multifocal FMD. Multifocal FMD patients might need to be followed less frequently but for a longer period because the recurrences would usually occur more than 5 years after PTRA. Thus, following up patients with multifocal FMD yearly, but for a longer period of at least 10 years or more, might be necessary. Although several studies have compared other clinical findings such as renal function, BP, median age at diagnosis, and affected vascular regions between the two types of FMD,[9–11] they have not discussed any difference in the period until recurrence between the types. Future trials should focus on the types of FMD and the age at diagnosis to investigate the long-term clinical outcomes of PTRA. As this paper presents only two case reports and a cross-sectional analysis of previous cases, more prospective research is necessary to determine the adequate follow-up period after PTRA.

Our report also suggests that patients of multifocal FMD with a younger age at onset may especially need to be observed for a longer period of time. A recent report showed that elderly patients with multifocal FMD were more likely to be asymptomatic and less likely to have had major vascular events or undergone a revascularization procedure than younger multifocal FMD patients [12]. Patients with multifocal FMD who develop symptoms at a younger age might have more significant and progressive RAS even after complete revascularization. Indeed, in patients 1 and 2, stenoses of the right renal artery developed in different segments after the previous lesion was revascularized by PTRA (Fig. 1). This indicates that the lesion had been gradually progressing and caused *de novo* stenoses. Many studies have been conducted to elucidate the etiology of FMD such as genetic, mechanical, or hormonal factors [13–15]. However, the pathological mechanism of FMD remains unclear. Unexpectedly, for the first time, the patients showed how multifocal FMD progresses over the years. This might help us to examine the etiology of the disease in the future, although we only observed just the morphological changes of the renal artery.

In conclusion, the current case reports and review suggest that the follow-up strategies for focal and multifocal FMD should be different. Patients with multifocal FMD should be followed up for more than 10 years, especially those in whom the onset was during their teenage years. These two cases prompted us to review other FMD cases retrospectively, because both patients had multifocal FMD and developed the disease at a young age. Our paper warrants the need for increased reporting of similar cases to support our findings and establish new guidelines for the follow-up period to detect recurrences of RVH in FMD after PTRA.

## Data Availability

The datasets used and analyzed in this study are available from the corresponding author on reasonable request.

## Abbreviations

**BP**: Blood pressure.
**CT**: Computed tomography.
**FMD**: Fibromuscular dysplasia.
**PSV**: Peak systolic velocity.
**PTRA**: Percutaneous transluminal renal angioplasty.
**RAR**: Renal/aorta ratio.
**RAS**: renal artery stenosis.
**RVH**: Renovascular hypertension.
